# Advances in Cotton Genomics, Genetics and Breeding

**DOI:** 10.3390/plants13182579

**Published:** 2024-09-14

**Authors:** Tianxu Zhang, Shuhui Wang, Jinhong Chen, Shuijin Zhu, Qianhao Zhu, Tianlun Zhao

**Affiliations:** 1 College of Agriculture and Biotechnology, Zhejiang University, Hangzhou 310058, China; 22316019@zju.edu.cn (T.Z.); jinhongchen@zju.edu.cn (J.C.); shjzhu@zju.edu.cn (S.Z.); 2 Hainan Institute, Zhejiang University, Sanya 572025, China; 12216020@zju.edu.cn; 3 CSIRO Agriculture and Food, GPO Box 1700, Canberra, ACT 2601, Australia

The cotton is an industrial crop of global significance, providing its fibers for the predominant textile material and its seed accumulating abundant oil and protein for other utilizations [1,2]. Given the context of land limitation and climate instability, it becomes urgent to accelerate the selective breeding of good-quality cotton varieties characterized by high-yield fibers, robust stress resistance, etc. [3,4,5] The boom of modern biotechnology shed light on this field, with revolutionary sequencing technology defying Moore’s law and remarkably effective gene-editing and function-verifying tools [6,7]. A vast array of the cotton genome assemblies, covering diverse varieties, enables the fine mapping and deep mining of genes underlying essential agronomy traits in conjunction with multi-omics data, further suggesting the comprehensive network [8,9,10,11]. However, molecular technology such as CRISPR/Cas9 offers a more solid foundation for elucidating the precise regulatory pathways and demonstrating profound potential in breeding [12,13]. Accordingly, the Special Issue of *Plants*, titled Advances in Cotton Genomics, Genetics and Breeding, aims to report progress on all aspects of cotton genomics, genetics, and breeding and hopes to fuel advancements in cotton breeding.

Plant height and branch number play a pivotal role in cotton development, both crucial for the mechanical harvesting and yield [14]. Li et al. [15] provide an in-depth understanding of these two agronomy traits in cotton. Through utilizing SNP-based markers within the RIL population derived from the variety CCRI70, they identified 69 QTLs for plant height (9 stable) and 63 for branch number (11 stable), with only one QTL for plant height reported before. Considering the expression pattern, enrichment result, and previous research, they elaborately present six possible key genes harbored in these stable QTLs. Not only does the study demonstrate the successful application of SNP-based markers in QTL mapping, but it also serves as valuable genetic resources for plant architecture in cotton breeding.

Fiber development has long been a central focus in cotton research, directly corresponding to the billion-dollar trade [3]. It can be divided into four continuous and overlapped stages: cell initiation, cell elongation, cell wall thickening, and maturation [16]. Li et al. [17] used RNA-seq data from different fiber development periods of two cotton varieties, Xin W 139 and *xin w 139*, which exhibited significant variance in fiber length and quality. The comparative analysis identified several genes that underpin the four stages of fiber development. Although considerable work remains to fully decipher the functions and mechanisms of these genes, their meticulous results serve as a useful reference for further research.

Liu et al. [18] determined the multiple biological functions of *GhZFP8* encoding an ethylene transcription factor. Their results indicated that the gene was actively involved in the trichome formation in Arabidopsis and fiber elongation in cotton and may also influence the phytohormone levels. Moreover, through DAP-Seq analysis, they also discovered its regulatory role in photosynthesis, signal transduction, and synthesis of biomass, all of which were intimately connected with fiber development.

From a systematical perspective, Zhai et al. [19] summarized the multifaceted mechanisms of fiber initiation and established comprehensive connections among them. They first described various spontaneous fiber initiation mutants contributing to the research. Then, they discussed the molecular mechanisms of the process from diverse perspectives, including phytohormones, transcription factors, sugar signals, small signal molecules, functional genes, non-coding RNAs, and histone modifications. Additionally, the crosstalk among these molecules was also illustrated, thus drawing a precise map of the fiber initiation, which offers significant insights on mechanisms and pathways.

The development of cotton also encounters threats from ion toxicity [20]. Nassarawa et al. [21] explored the effectiveness of zinc sulfate and ZnO nanoparticles in relieving Boron toxicity in cotton. Their controlled experiments demonstrated that both zinc sulfate and ZnO nanoparticles could reduce boron toxicity, particularly the latter. Furthermore, they also tried to provide a theoretical basis for such an anti-stress process utilizing RNA-seq and discovered that these zinc-based treatments enhance the photosynthesis and antioxidants suppressed by boron toxicity and regulate the mineral nutrients to reduce the boron contents themselves. The research fills the gap between the application of exogenous substances and the mitigation of boron toxicity in cotton, contributing to the understanding of the mechanisms of zinc in alleviating boron toxicity.

Recent emerging and robust techniques have advanced the deciphering of cotton gene functions [5,12]. Tian et al. [22] detailed the use of virus-induced gene silencing (VIGS) in cotton functional genomic studies. They carefully described the history and application methods of VIGS. They then demonstrated the successful application of this technique in cotton gene functions research covering biotic and abiotic stress, nutrition, and reproductive development in detail. Moreover, they highlighted the limitation of the VIGS for its sharply decreasing effectiveness after flowering and discussed the potential improvement to the method. The review provides us with a specific guide to using VIGS in cotton research.

The articles featured in this Special Issue cover a wide range of cotton genomic research, which significantly underscores the precision and speed that modern genetic tools bring to traditional breeding. We hope that these articles inspire the readers, thereby facilitating more efficient approaches for cotton breeding targeting higher resistance, yield, and quality.
